# Bile from Patients with Primary Sclerosing Cholangitis Contains Mucosal-Associated Invariant T-Cell Antigens

**DOI:** 10.1016/j.ajpath.2021.12.008

**Published:** 2022-01-19

**Authors:** Laura Valestrand, Fei Zheng, Simen H. Hansen, Jonas Øgaard, Johannes R. Hov, Niklas K. Björkström, Tom H. Karlsen, Xiaojun Jiang, Espen Melum

**Affiliations:** ∗ Norwegian PSC Research Center, Division of Surgery, Inflammatory Diseases and Transplantation, Oslo University Hospital, Oslo, Norway; † Research Institute of Internal Medicine, Oslo University Hospital, Oslo, Norway; ‡ Institute of Clinical Medicine, University of Oslo, Oslo, Norway; § Section of Gastroenterology, Division of Surgery, Inflammatory Diseases and Transplantation, Oslo University Hospital, Oslo, Norway; ¶ Center for Infectious Medicine, Department of Medicine Huddinge, Karolinska Institutet, Karolinska University Hospital, Stockholm, Sweden; ‖ Hybrid Technology Hub-Centre of Excellence, Institute of Basic Medical Sciences, Faculty of Medicine, University of Oslo, Oslo, Norway

## Abstract

Primary sclerosing cholangitis (PSC) is associated with altered microbiota of the gut and bile. Mucosal-associated invariant T (MAIT) cells, enriched in human liver, uniquely recognize microbial-derived metabolites. This study aimed to determine whether bile from patients with PSC contains antigens activating MAIT cells. Bile was collected at the time of liver transplantation from patients with PSC (*n* = 28). The bile samples were either directly incubated with peripheral blood mononuclear cells from healthy donors or with antigen-presenting cells followed by co-culture with peripheral blood mononuclear cells. MAIT cell activation was assessed by flow cytometry. An anti-MR1 antibody was used to determine whether the activation was major histocompatibility complex class I–related protein (MR1) restricted. Biliary microbiota profiles were generated using 16S rRNA amplicon sequencing, and the abundance of the bacterial gene *ribD* was predicted. Eight of 28 bile samples could activate MAIT cells. This activation was partly MR1-dependent in five of eight bile samples. Microbial DNA was detected in 15 of 28 bile samples, including the five bile samples leading to MR1-dependent activation. A higher abundance of the *ribD* gene expression in the group of bile samples that could activate MAIT cells was predicted on the basis of the 16S sequencing. In co-culture experiments, cholangiocytes could take up and present biliary antigens to MAIT cells. These findings suggest a pathophysiological pathway in PSC connecting the immune system and the microbiome.

Primary sclerosing cholangitis (PSC) is a chronic liver disease, characterized by bile duct inflammation, progressive fibrosis, and a strong association with inflammatory bowel disease (IBD), seen in up to 80% of the patients.^1^^,^^2^ There is no established pharmacologic treatment, and liver transplantation remains the only curative treatment.^3^^,^^4^ A range of genetic studies have positioned PSC as an autoimmune disease, but major aspects of PSC pathophysiology remain largely unknown.^1^^,^^5^ The gut microbiome plays a role in PSC pathophysiology, as evidenced by a distinct microbiome in patients with PSC, and a reduction of alkaline phosphatase levels on treatment with antibiotics.^6^, ^7^, ^8^ Classic bile culture and next-generation sequencing (16S rRNA sequencing) studies have demonstrated an altered biliary microbial community in patients with PSC, with reduced richness compared with disease controls, and altered presence of specific bacteria.^9^, ^10^, ^11^ In a gnotobiotic mouse study, the PSC-associated bacteria *Klebsiella pneumoniae* was found to contribute to disrupted intestinal barrier function, and inoculation with PSC-derived microbiota of gnotobiotic mice induced a hepatic type 17 helper T-cell response that increased the susceptibility of the mice to hepatobiliary disease progression.^12^ Along with disease progression in PSC patients, bacterial infections of the bile ducts are a common complication and recurrent biliary infections can in themselves be an indication for liver transplantation.^13^ Collectively, these studies indicate that the bile microbiome itself, either as a primary factor or as an important modulator, is involved in the pathophysiology of PSC.

Mucosal-associated invariant T (MAIT) cells are a subset of innate-like T cells that represent up to 45% of liver lymphocytes.^14^^,^^15^ MAIT cells are characterized by an invariant T-cell receptor (TCR) α chain Vα7.2-Jα33 in humans and Vα19-Jα33 in mice,^16^ and display an effector memory phenotype with rapid response to stimulation with release of proinflammatory or cytotoxic cytokines such as interferon-γ, IL-17, and granzyme B (GrB).^15^^,^^17^^,^^18^ MAIT cells uniquely recognize microbial-derived vitamin B metabolites presented by major histocompatibility complex class I–related protein (MR1).^19^^,^^20^ Among these, 5-(2-oxopropylideneamino)-6-d-ribitylaminouracil (5-OP-RU), a riboflavin synthesis metabolite, is reported to be a particular potent antigen.^21^ The *ribD* gene encodes one of the key riboflavin synthase enzymes that is highly expressed in some bacteria, suggesting that these bacteria produce vitamin B metabolites and thereby represent a source of potential MAIT cell antigens.^22^

MAIT cells have a role in a range of different diseases, varying from autoimmunity^23^, ^24^, ^25^ to cancers^26^, ^27^, ^28^ and infectious diseases.^29^ Liver MAIT cells associate with inflammation and fibrosis in primary biliary cholangitis, hepatitis C virus infection, alcohol-related liver disease, autoimmune hepatitis, nonalcoholic steatohepatitis, and PSC, suggesting broad effects in development of liver pathologies.^30^, ^31^, ^32^, ^33^, ^34^, ^35^ A link between MAIT cells and fibrosis has been proposed by activation of hepatic myofibroblasts and hepatic stellate cells through IL-17 production.^31^^,^^32^

Cholangiocytes, the cells lining the bile ducts, can present MR1 restricted antigens to MAIT cells, which are preferably localized close to intrahepatic bile ducts.^30^ We therefore hypothesized that bile contains potential MAIT antigens that can be processed by cholangiocytes and presented to MAIT cells to initiate or modulate the immune response in inflammatory bile duct diseases.

## Materials and Methods

### Patient Bile Samples and Peripheral Blood from Healthy Donors

Bile was collected from the gallbladder of patients directly after liver transplantation due to PSC (*n* = 28), alcohol-related liver disease (*n* = 4), autoimmune hepatitis (*n* = 2), or hemochromatosis (*n* = 1). A sterile scalpel was used to cut a small opening in the gallbladder, and a minimum of 3 mL of bile was aspirated into sterile tubes with a sterile 20-mL syringe, stored, and aliquoted on ice until longtime storage at −80°C. Written informed consent was obtained from all study participants. In accordance with the Declaration of Helsinki, ethical approval was obtained from the Regional Committees for Medical and Health Research Ethics of South East Norway (reference numbers 2012-286 and 2016-1540). Buffy coats from healthy donors were obtained from Oslo University blood bank, and the usage was approved by Regional Committee for Medical and Health Research Ethics of South East Norway (reference number S-05172).

### Clinical Characterization

For clinicopathologic characterizations of included patients, the following indexes were calculated:

Model for End-Stage Liver Disease Sodium score^36^ was calculated on the basis of information on dialysis treatment the preceding week before transplantation and the laboratory values for creatinine, bilirubin, and international normalized ratio at the time of transplantation. Aspartate Aminotransaminase to Platelet Ratio Index test^37^ was calculated on the basis of the aspartate aminotransaminase values and platelet count at the timepoint of transplantation. Child Pugh Score^38^ was calculated on the basis of presence of ascites and encephalopathy and the laboratory values for international normalized ratio, bilirubin, and albumin at the timepoint of transplantation. Fibrosis-4 Index for liver fibrosis^39^ was calculated on the basis of age, aspartate aminotransaminase, alanine aminotransaminase, and platelet count at the timepoint of transplantation.

### Cell Isolation and Cell Culture

Peripheral blood mononuclear cells (PBMCs) were separated from buffy coats using Ficoll Paque Plus (GE Healthcare Life Sciences, Uppsala, Sweden). The collected PBMCs were aliquoted in a cell cryopreservation media (Merck KGaA, Darmstadt, Germany) and stored in liquid nitrogen. For experiments, PBMCs were thawed, washed, and maintained in RPMI 1640 medium (Lonza, Basel, Switzerland) supplemented with 2 mmol/L l-glutamine (Merck KGaA), 10% fetal bovine serum (Thermo Fisher Scientific, Waltham, MA), 1% Gibco Antibiotic-Antimycotic (10,000 units/mL of penicillin, 10,000 μg/mL of streptomycin, and 25 μg/mL of Amphotericin B; Thermo Fisher Scientific). A human monocyte cell line (THP1) was maintained in supplemented media as described for the PBMCs, and a previously immortalized human cholangiocyte cell line (H69) was cultured in conditioned medium as previously described.^40^, ^41^, ^42^ Both cell lines were maintained in 37°C incubators with 5% CO_2,_ and the PBMCs were cultivated for at least 1 hour before application in experiments.

### *Escherichia coli* Culture and Fixation

*Escherichia coli* (DH5α; Thermo Fisher Scientific) was cultured overnight at 37°C in Luria-Bertani broth (Sigma-Aldrich, St. Louis, MO), then counted by standard plate-counting methods, aliquoted in a solution made by 50% glycerol and 50% fetal calf serum, and stored at −80°C. To fixate the *E. coli*, aliquots were thawed and washed in phosphate-buffered saline (PBS), then incubated for 5 minutes at room temperature in 1% formaldehyde (HistoLab, Gothenburg, Sweden) and vortexed before washing and resuspension in PBS in different dilutions.

### 5-OP-RU Preparation

5-Amino-6-d-ribitylaminouracil (5-A-RU; MuseChem, Fairfield, NJ) was stored at 4°C in solid form until dissolved in sterile and distilled water, and frozen at −80°C in 5 mmol/L stock solutions. Fifteen minutes before application in experiments, the solid chemical was mixed with methylglyoxal (Sigma-Aldrich) and used in the indicated doses.

### Immunofluorescence Staining of MR1 on H69 Cells

A total of 100,000 H69 cells in 500 μL conditioned medium were seeded on eight-chamber slides (Thermo Fisher Scientific) before overnight incubation in a 37°C incubator with 5% CO_2_. The slides were then washed 2 × 1 minute in cold PBS before 15-minute fixation with formaldehyde 4% (Sigma-Aldrich) and permeabilized for 5 minutes with 0.2% Triton X-100 (Sigma-Aldrich) at room temperature. The slides were then blocked with 1% bovine serum albumin and 0.1% Tween 20 in PBS for 15 minutes before washings and staining with primary anti-MR1 antibody (catalog number 13260-1-AP; Proteintech, Rosemont, IL) or an isotype control antibody (catalog number 30000-0-AP; Proteintech) for 1 hour at room temperature. The slides were then washed and incubated for 1 hour with Alexa Fluor 488 goat anti-rabbit secondary antibody (catalog number A11008; Thermo Fisher Scientific) before washings and mounting with ProLong Gold antifade with DAPI (Thermo Fisher Scientific) and coverslips. After an overnight incubation at room temperature in the dark, images were acquired with an Eclipse e400 Fluorescent Microscope (Nikon, Tokyo, Japan) with a DS-Fi1 camera (Nikon) controlled by NIS-Element BR 3.10 software (Nikon) with identical exposure times and camera settings.

### MAIT Cell Activation Assay

PBMCs were plated in 96 round-bottom plates (5 × 10^5^ cells in 200 μL medium per well in triplicates) and incubated in supplemented medium with bile (diluted 1:200), fixed *E. coli* (250 colony-forming units/cell), or PBS for 24 hours. In experiments with multiple PBMC donors, the PBMCs were plated as singlets instead of triplicates and they were incubated with bile for 48 hours; otherwise the protocol was identical to experiments with one PBMC donor. In experiments with blocking of MR1-mediated antigen presentation, 20 μg/mL of the monoclonal anti-MR1 antibody clone 26.5 (BioLegend, San Diego, CA) was added 30 minutes before incubation with bile or fixed *E. coli*. Intracellular cytokines were analyzed by flow cytometry after 6 hours of culture with brefeldin A and monensin (eBioscience, San Diego, CA).

### Co-Culture Activation Assay

In co-culture assays, THP1 or H69 cells were used as antigen-presenting cells (APCs) and seeded in 96-well flat-bottomed plates for H69 cells and round-bottomed plates for THP1 cells (8 × 10^4^ cells per well) followed by overnight incubation. Bile (diluted 1:200), fixed *E. coli* (250 colony-forming units/cell), or PBS was then added, followed by 24 hours of incubation. Antigens not taken up by the APCs were washed off with cell culture medium, followed by addition of PBMCs and 24 hours of incubation. In experiments with blocking of MR1-mediated antigen presentation, 20 μg/mL of an MR1-blocking antibody (clone 26.5) was added 30 minutes before incubation with PBMCs. Intracellular cytokines were analyzed by flow cytometry after 6 hours of culture with brefeldin A and monensin (eBioscience).

### Antibodies and Flow Cytometry

The following antibodies against human epitopes were used: CD3 [1:200; clone HIT3a; phycoerythrin (PE); catalog number 300308], TCR Vα7.2 (1:100; 3C10; APC; catalog number 351708), CD161 [1:100; HP-3G10; fluorescein isothiocyanate (FITC); catalog number 339906], CD19 (1:200; HIB19; APCCY7; catalog number 302218), CD69 (1:200; FN50; PECY7; catalog number 310912), granzyme B (1:200; QA16A02; PercpCy5.5; catalog number 372212), and viability dye (1:100; Zombie NIR; catalog number 423106), all purchased from BioLegend. Human MR1 5-OP-RU (1:800, PE) and MR1 6-formylpterin (6-FP, PE) (1:800) tetramers were kindly provided by the NIH Tetramer Core. Cell surface markers were stained using directly conjugated antibodies for 30 minutes at 4°C in PBS with 2% fetal calf serum, and dead cells were excluded using a live/dead fixable viability dye (Zombie NIR). For staining of intracellular markers, cells were fixed for 45 minutes using BD Fixation/Permeabilization Kit (BD Biosciences, San Jose, CA) and washed once in Perm/Wash before 45 minutes of staining with intracellular monoclonal antibodies in Perm/Wash followed by the last wash with Perm/Wash and data acquisition. Flow cytometric analysis was performed using a BD FACS Verse flow cytometer (BD Biosciences), and results were analyzed in Flow Jo version 10.1 (BD Life Science, San Jose, CA).

### 16S rRNA Gene Sequencing and Analysis

Microbial DNA from bile samples was extracted using the QIAamp DNA mini kit (Qiagen, Hilden, Germany), as previously described.^43^^,^^44^ The hypervariable regions V3 and V4 of the prokaryotic 16S rRNA gene were amplified using the 319F-806R primer pair and a dual-indexing approach with barcoded primers and Phusion High-Fidelity PCR master mix with HF buffer (Thermo Fisher Scientific).^45^ 16S rRNA sequencing on the Illumina MiSeq (Illumina Inc., San Diego, CA) platform and data analysis were performed as described previously.^44^ In short, paired-end reads were filtered for Illumina Universal Adapters and PhiX, demultiplexed, quality trimmed, and merged. Denoising to amplicon sequence variants and taxonomic classification were performed using the Deblur Plugin^46^ in the Quantitative Insights Into Microbial Ecology 2 platform version 2019.7.^47^ In each bile sample, taxa with number of reads of <100 were discarded. There were no detectable levels of bacteria in the negative controls, and so no identified contaminants were removed from the data set before further analyses were performed. PICRUSt2 analysis was run on unfiltered bile samples to predict the abundance of the *ribD* gene in the sequenced bacteria compared against a published gene database.^48^

### Statistical Analysis

All values are presented as means ± SEM unless otherwise stated. Statistical significance was evaluated using *t*-test for variables meeting criteria of normal distribution. For experiments where multiple comparisons were included, one-way analysis of variance was used followed by correction for multiple testing using the Bonferroni method. To evaluate statistical significance for categorical data, a Fisher exact test was applied. For evaluating correlations between clinicopathologic scores and presence of MAIT antigens or *ribD* abundance, a Pearson *r* test was performed. *P* < 0.05 was considered statistically significant. Statistical tests were performed using the Prism GraphPad software version 8.0 (Graphpad Software Inc., La Jolla, CA).

## Results

### Bile from Patients with PSC Activates MAIT Cells

**Figure 1:**
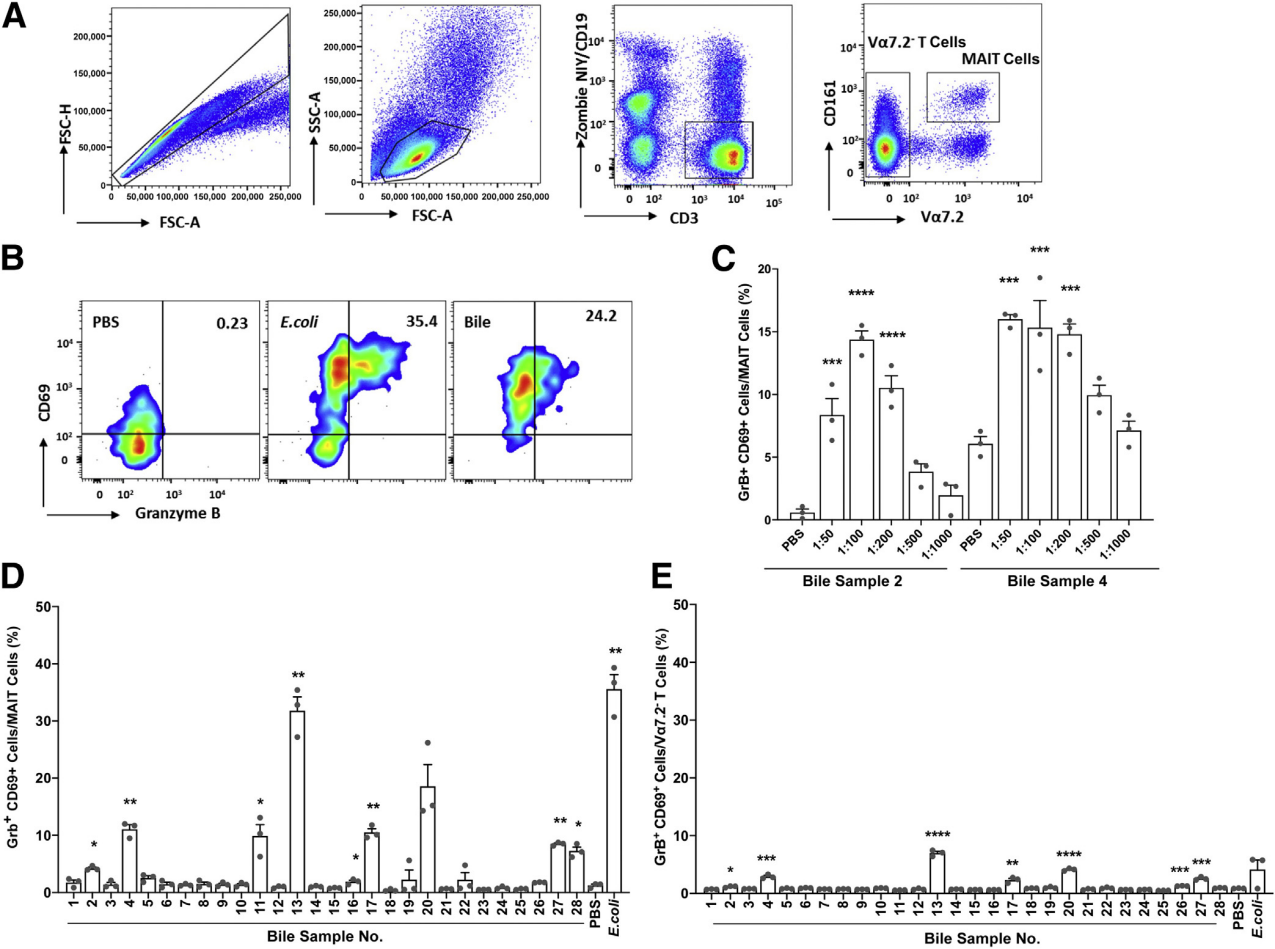
Mucosal-associated invariant T (MAIT) cells are activated by antigens in bile from patients with primary sclerosing cholangitis (PSC). **A:** Representative flow plots show the gating strategy for identifying human MAIT cells; CD161^+^ T-cell receptor Vα7.2^+^ T cells within peripheral blood mononuclear cells (PBMCs) after excluding B cells and dead cells (CD19 and Zombie NIR) and duplicates. Gating strategy for Vα7.2^−^ T cells is also shown. **B:** Representative flow plots of CD69^+^ granzyme B^+^ (GrB^+^) MAIT cells, after incubation with phosphate-buffered saline (PBS), fixed *Escherichia coli*, or bile and PBMCs. **C:** Bar plots showing the percentage of CD69^+^ GrB^+^ MAIT cells within PBMCs after incubation with bile sample numbers 2 and 4 in serial dilutions. **D** and **E:** Bar plots showing the percentage of CD69^+^ GrB^+^ MAIT cells (**D**) or CD69^+^ GrB^+^ Vα7.2^−^ T cells (**E**) after stimulation with 28 bile samples (diluted 1:200) that were collected from the gallbladder from patients with PSC at the time of liver transplantation. Fixed *E. coli* is used as positive control, and PBS is used as negative control. **C**–**E**: Experiments in were performed with triplicates. **D** and **E:** Representative results from one of three independent experiments with three healthy donors are shown. **C:** Representative results from one of two experiments with two healthy donors for bile 2 and one donor for bile 4 are shown. **C**–**E:** Statistical significance was evaluated by *t*-test (**D** and **E**) and one-way analysis of variance, followed by correction for multiple testing using the Bonferroni method within each bile sample in serial dilutions, including the PBS control (**C**). **C**–**E:***P* indicates the results from significance testing between individual bile samples compared with PBS. All data are presented as means ± SEM (**C**–**E**). ∗*P* < 0.05, ∗∗*P* < 0.01, ∗∗∗*P* < 0.001, and ∗∗∗∗*P* < 0.0001. FSC, forward scatter; SSC, side scatter.

**Supplemental Figure S1:**
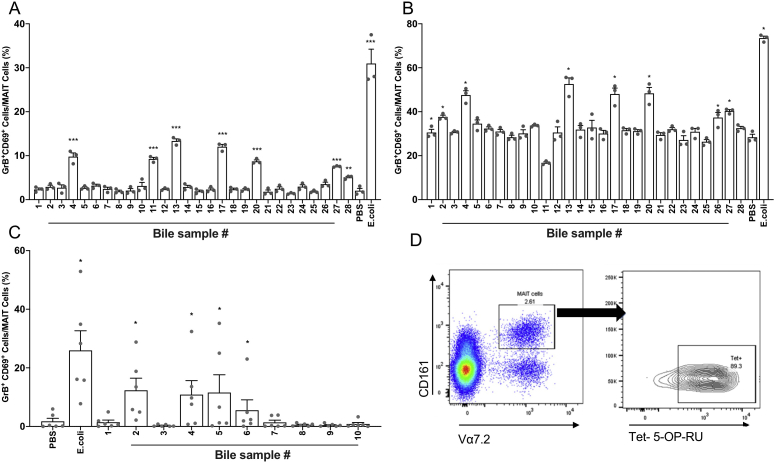
Activation of mucosal-associated invariant T (MAIT) cells by biliary antigens. **A** and **B:** A total of 28 bile samples were incubated with peripheral blood mononuclear cells (PBMCs) from two different healthy donors, and the bar plots show percentage of CD69^+^ and granzyme B^+^ (GrB^+^) MAIT cells. **C:** PBMCs from six different healthy donors were incubated with 10 bile samples or fixed *Escherichia coli*. The graphs show the percentage of CD69^+^ and granzyme B^+^ MAIT cells. **D:** Confirmation of the antibody-based gating strategy for MAIT cells using the 5-(2-oxopropylideneamino)-6-d-ribitylaminouracil (5-OP-RU) tetramer (1:800). All experiments were performed with triplicates, except in **C**. Statistical significance was evaluated by *t*-test. *P* indicates the results from significance testing between individual bile samples compared with phosphate-buffered saline (PBS). Results are presented as means ± SEM (**A**–**C**). ∗*P* < 0.05, ∗∗*P* < 0.01, and ∗∗∗*P* < 0.001.

To investigate the presence of potential MAIT cell antigens, bile from 28 patients with PSC (Table 1) was screened using PBMCs from healthy donors (Figure 1A, two additional donors are shown in Supplemental Figure S1). The percentages of GrB^+^ and CD69^+^ MAIT cells were analyzed by flow cytometry (gating strategies in Figure 1, A and B, and Supplemental Figure S1) with *E. coli* as a positive control. Incubation with eight of the bile samples led to the activation of MAIT cells, as measured by increased CD69 and GrB expression (Figure 1, B and D, and Supplemental Figure S1). To evaluate the potency of the antigens, a serial-dilution experiment was performed for two of the bile samples demonstrating a clear dose-response relationship (Figure 1C). Because 20 of 28 bile samples did not activate MAIT cells and because MAIT cell response induced by the different bile samples was highly variable, it is unlikely that the activation of MAIT cells results from a physiological constituent in bile.

**Table 1:** Clinical Characteristics of Bile Sample Donors with PSC

								Variables at time of transplantation			
BileSample no.	Sex	Diagnosis	MELD-Na score	Liver cirrhosis	UDCA/AB	Comorbidities	ERC	Bilirubin,mg/dL	ALT,U/L	ALP,U/L	CRP,mg/L
1	F	PSC	18	Yes	No/yes	CD	No	5.0	37	141	24
2	M	PSC	8	No	No/yes		Yes	1.4	41	240	10
3	M	PSC	10	Yes	No/no	HT, adrenal insufficiency, UC	No	1.4	104	238	15
4	M	PSC	13	No	Yes/yes	Hypothyroidism, UC	No	4.6	120	505	50
5	M	PSC	9	No	Yes/no	UC, celiac disease, arthralgia	No	2.2	264	528	4.6
6	F	PSC	12	Yes	No/no		Yes	3.2	82	325	1.7
7	F	PSC	22	Yes	Yes/no	UC	No	14.5	89	281	23
8	M	PSC	7	Yes	No/no		No	0.5	26	167	12
9	F	PSC, HCC	6	Yes	Yes/no	UC, colectomy	No	0.9	44	142	2
10	F	PSC	6	No	No/no	UC, colectomy	Yes	0.4	31	69	1.1
11	M	PSC	17	No	No/yes		No	4.2	87	113	22
12	M	PSC	17	Yes	No/no	Ileoanal pouch, UC, colectomy	No	2.4	171	473	11
13	M	PSC	22	Yes	No/yes	UC	Yes	17.7	103	724	15
14	F	PSC	7	No	Yes/no	Asthma	Yes	0.9	64	193	5.7
15	M	PSC	7	Yes	Yes/no	UC	No	1.2	128	344	3.1
16	M	PSC	19	Yes	Yes/no	Indetermined colitis	No	5.3	100	665	46
17	M	PSC	15	Yes	No/no	UC	Yes	5.1	127	722	14
18	M	PSC	6	Yes	Yes/no	WPW syndrome, UC	Yes	0.6	229	337	3.5
19	F	PSC	6	No	No/no	UC	Yes	0.4	35	71	2.8
20	M	PSC	8	No	No/no	UC	Yes	0.8	119	255	1.1
21	M	PSC	13	Yes	No/no	UC	No	2.1	323	162	7.8
22	M	PSC	15	No	No/no	UC	Yes	10.6	339	565	17
23	M	PSC	6	No	No/yes	UC	Yes	0.6	61	407	72
24	M	PSC	7	No	No/no		No	0.4	36	68	1.2
25	M	PSC	6	Yes	No/yes	HCM	Yes	0.6	97	392	1.9
26	M	PSC, HCC	18	Yes	No/no	UC	No	4.0	44	177	73
27	M	PSC	15	Yes	Yes/yes	CD, gastric ulcer	No	4.2	72	203	22
28	M	PSC	15	Yes	Yes/no	IgA nephritis, UC, HCM	Yes	7.1	242	312	20

The presence of liver cirrhosis in the explanted liver was evaluated by a liver pathologist. Biochemistry values represent the values just before liver transplantation, and whether ERC had been performed the last 6 months before liver transplantation was registered.

F, female; M, male; AB, antibiotics; ALP, alkaline phosphatase; ALT, alanine aminotransferase; CD, Crohn disease; CRP, C-reactive protein; ERC, endoscopic retrograde cholangiography; HCC, hepatocellular carcinoma; HCM, hemochromatosis; HT, hypertension; MELD-Na, Model for End-Stage Liver Disease Sodium; PSC, primary sclerosing cholangitis; UC, ulcerative colitis; UDCA, ursodeoxycholic acid; WPW, Wolff-Parkinson-White.

**Supplemental Figure S2:**
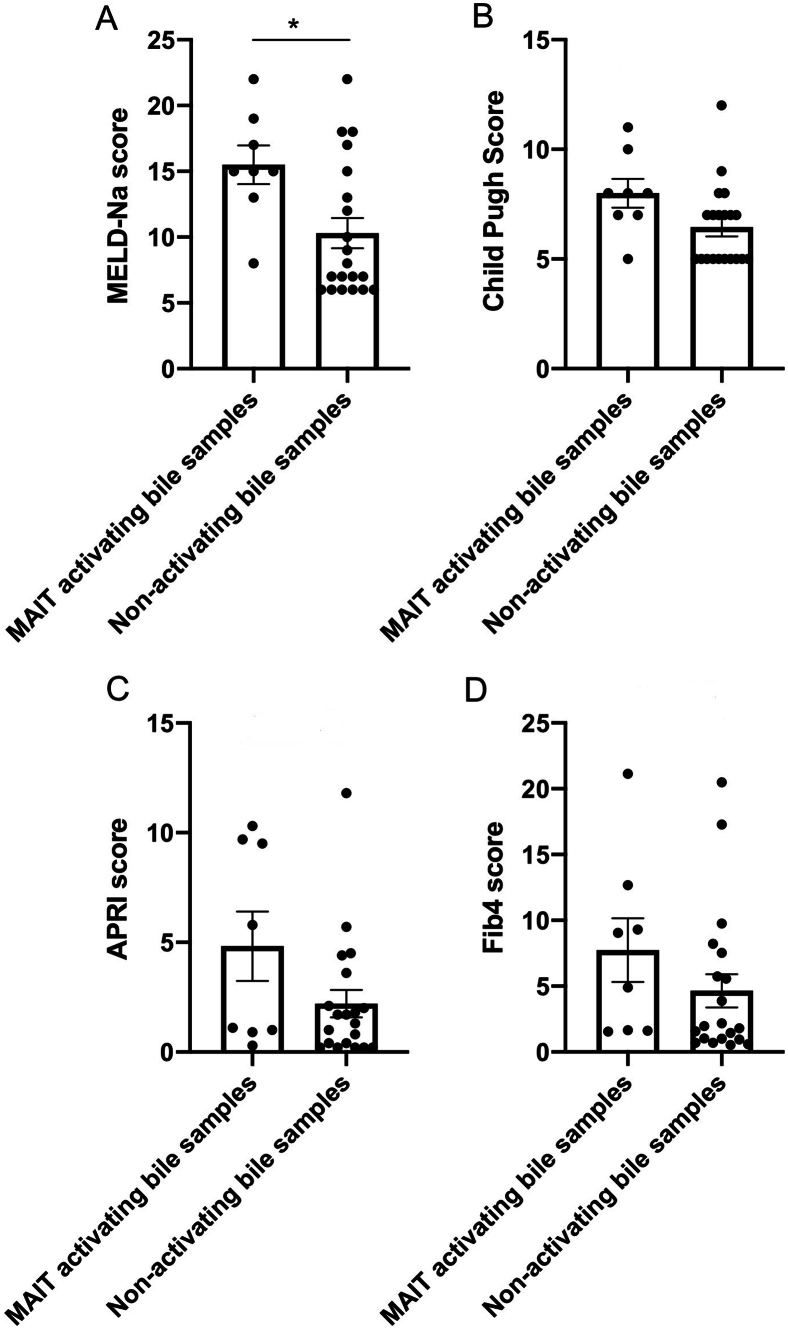
Difference in clinicopathologic scores between primary sclerosing cholangitis patients with mucosal-associated invariant T (MAIT) cell activating and nonactivating bile samples. Model for End-Stage Liver Disease Sodium (MELD-Na) score distribution (**A**), Child Pugh Score (**B**), Aspartate Aminotransaminase to Platelet Ratio Index (APRI) test distribution (**C**), and Fibrosis-4 (Fib4) Index for liver fibrosis distribution (**D**). Statistical significance was evaluated by *t*-test. ∗*P* < 0.05.

Expression levels of CD69 and GrB on the Vα7.2^−^ T cells (ie, non-MAIT cells) were considerably lower than those observed for MAIT cells, indicating that the activation of MAIT cells was specific and not due to a general immune cell activation (Figure 1E). Patients with PSC with MAIT cell activating bile demonstrated a higher Model for End-Stage Liver Disease Sodium score than patients with PSC without MAIT cell activating bile (*P* = 0.016) and a trend towards higher scores for three other clinicopathologic indexes: Aspartate Aminotransaminase to Platelet Ratio Index test (*P* = 0.072), Child Pugh Score (*P* = 0.056), and Fibrosis-4 Index (*P* = 0.23) (Supplemental Figure S2).

### Professional Antigen-Presenting Cells and Cholangiocytes Take Up Antigens and Activate MAIT Cells

**Figure 2:**
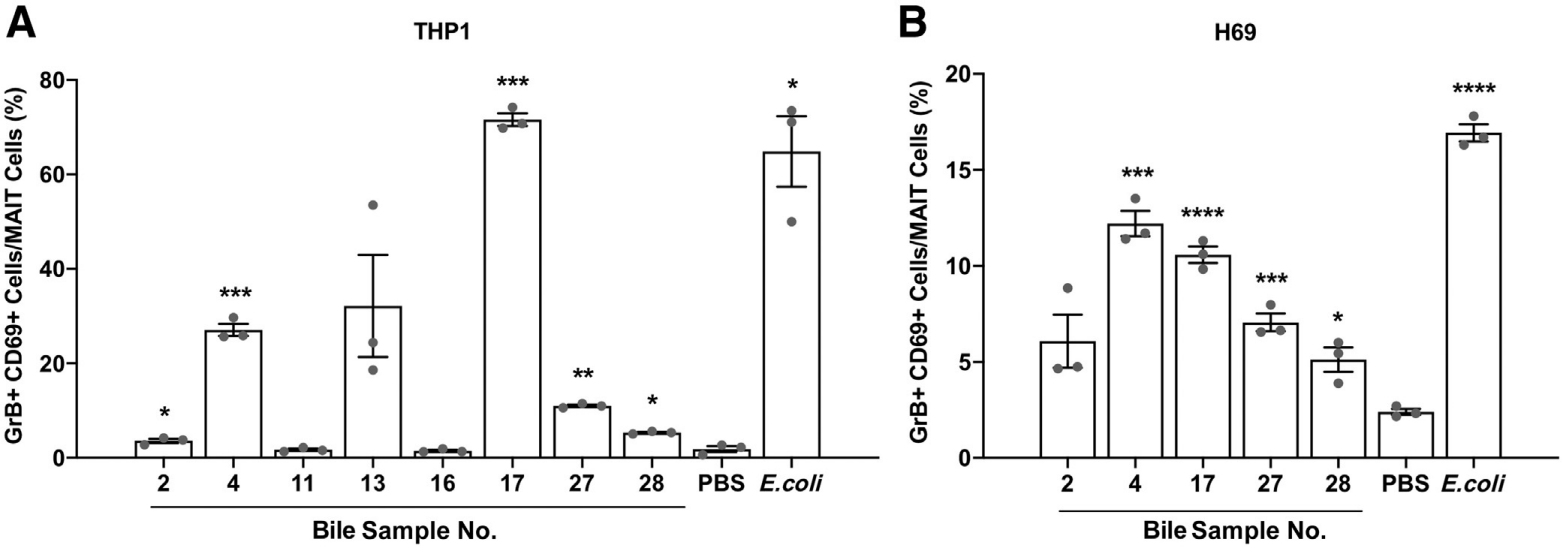
Antigen-presenting cells take up biliary antigens and activate mucosal-associated invariant T (MAIT) cells. **A:** Percentages of CD69^+^ granzyme B^+^ (GrB^+^) MAIT cells within peripheral blood mononuclear cells after co-culture with THP1 as antigen-presenting cells that were preloaded with biliary antigen from the eight bile samples activating MAIT cells. **B:** Bar plots showing the percentages of CD69^+^ GrB^+^ MAIT cells in co-culture with a human cholangiocyte cell line H69 preloaded with the five bile samples that the THP1 cells in **A** could present to and activate MAIT cells. All experiments were performed in triplicates. **A:** Representative results from one of three independent experiments using three healthy donors are shown. **B:** Representative results from one of two independent experiments with the same healthy donor are shown. Statistical significance was evaluated by *t*-test. *P* indicates the results from significance testing between individual bile samples compared with phosphate-buffered saline (PBS). All data are presented as means ± SEM (**A** and **B**). ∗*P* < 0.05, ∗∗*P* < 0.01, ∗∗∗*P* < 0.001, and ∗∗∗∗*P* < 0.0001.

**Supplemental Figure S3:**
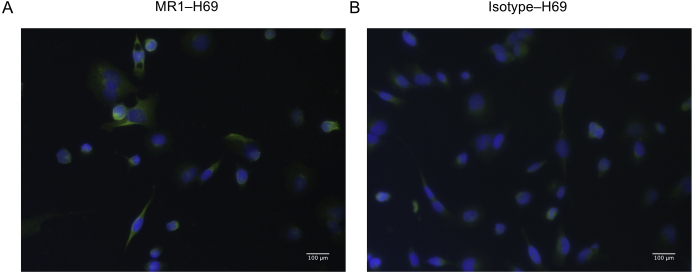
Major histocompatibility complex class I–related protein (MR1) immunofluorescence staining of H69 cells. **A:** Staining with an anti-MR1 antibody. **B:** Control staining with an isotype antibody. Representative images of from two independent experiments are shown. Scale bar = 100 μm (**A** and **B**).

**Supplemental Figure S4:**
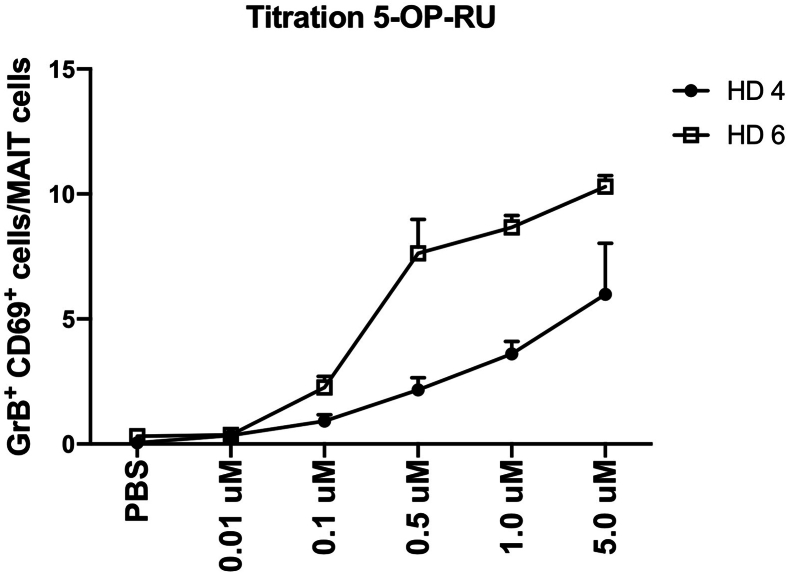
Serial dilutions of 5-(2-oxopropylideneamino)-6-d-ribitylaminouracil (5-OP-RU) incubated with the human cholangiocyte cell line H69 and peripheral blood mononuclear cells (PBMCs). The percentage of CD69^+^ and granzyme B^+^ (GrB^+^) mucosal-associated invariant T (MAIT) cells among PBMCs is shown. HD, healthy donor; PBS, phosphate-buffered saline.

To investigate whether MAIT antigens in bile could be taken up and presented by APCs and subsequently activate MAIT cells, a co-culture assay with THP1 cells, a cell type that has previously been shown to take up and present MR1 antigens, was used.^49^^,^^50^ Eight bile samples were used, which were able to activate MAIT cells in the aforementioned experiments. Of these, five bile samples led to activation of MAIT cells after incubation with APCs followed by addition of MAIT cells within PBMCs (Figure 2A). Next, whether human cholangiocytes could take up these potential antigens from bile and activate MAIT cells was examined. Studies first confirmed that the cholangiocyte cell line H69 expressed MR1 by immunofluorescence staining (Supplemental Figure S3) and that the cells were capable of presenting 5-OP-RU, leading to activation of MAIT cells in a dose-dependent manner (Supplemental Figure S4). Five bile samples that activated MAIT cells after preincubation with the THP1 cell line were used, and four of these samples led to activation of MAIT cells in the assay using H69 cells as APCs (Figure 2B).

### MAIT Cell Activation by Antigens in Bile Is Partially MR1-Dependent

**Figure 3:**
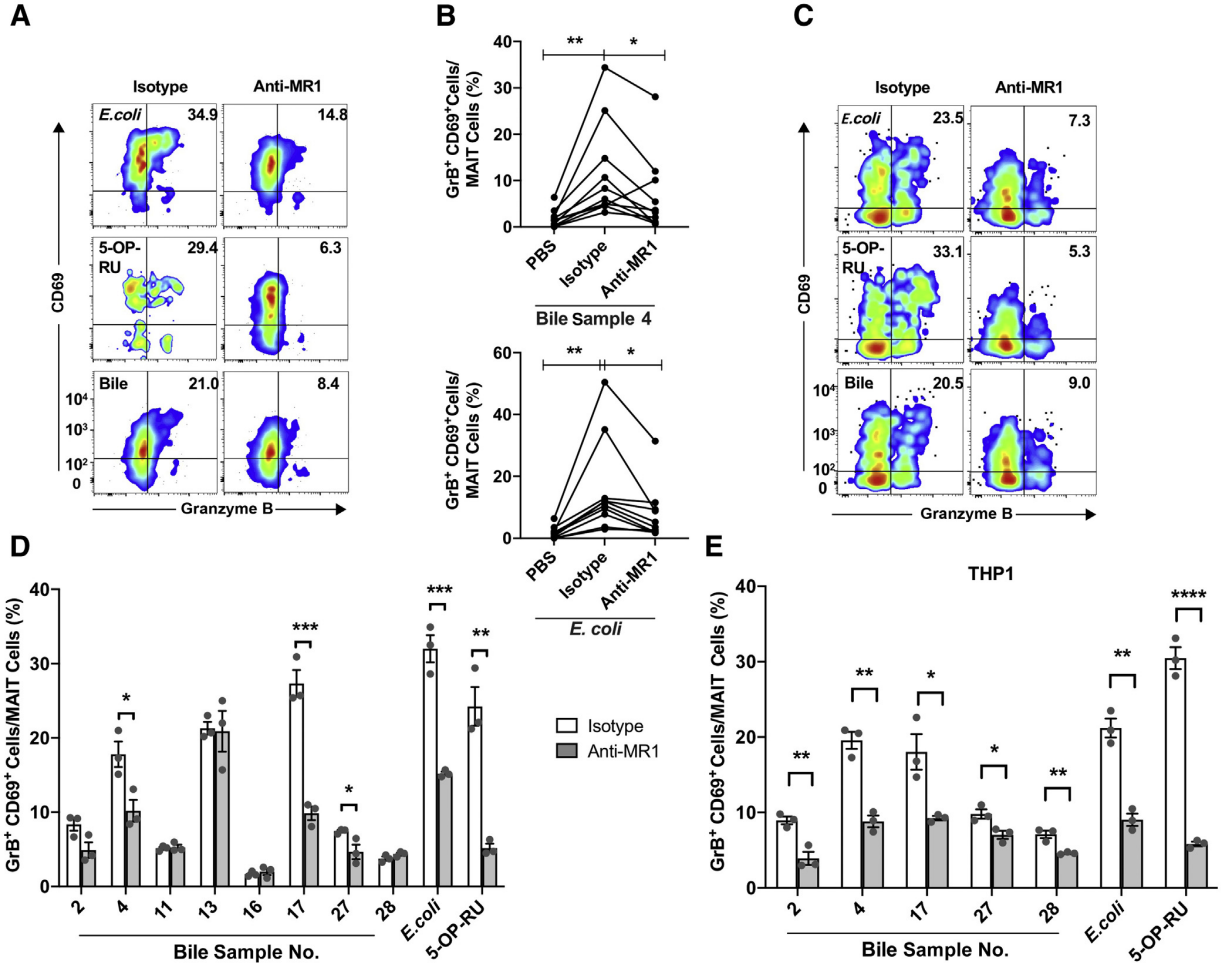
Mucosal-associated invariant T (MAIT) cell activation is partially major histocompatibility complex class I–related protein (MR1) restricted. **A:** Representative flow plots showing CD69^+^ granzyme B^+^ (GrB^+^) activated MAIT cells within peripheral blood mononuclear cells (PBMCs) after incubation with *Escherichia coli*, 5-(2-oxopropylideneamino)-6-d-ribitylaminouracil (5-OP-RU), or bile and an anti-MR1 antibody or an isotype control. **B:** Graph showing percentage of CD69^+^ GrB^+^ activated MAIT cells incubated with an anti-MR1 antibody or an isotype control incubated together with PBMCs from 10 different healthy donors and *E. coli* or bile sample 4. **C:** Representative flow plots showing CD69^+^ GrB^+^ activated MAIT cells after incubation with THP1 cells as antigen-presenting cells that had been preincubated with bile, fixed *E. coli*, or 5-OP-RU and an anti-MR1 antibody or an isotype control. **D:** Bar plots showing the percentage of CD69^+^ GrB^+^ activated MAIT cells within PBMCs incubated with an anti-MR1 antibody or an isotype control. **E:** Bar plots showing the percentage of CD69^+^ GrB^+^ activated MAIT cells within PBMCs after incubation with THP1 cells preincubated with bile, fixed *E. coli*, or 5-OP-RU and the anti-MR1 antibody or an isotype control. All experiments were performed in triplicates, except in **B**. **A**–**E:** Representative results from two independent experiment (**A**, **B**, and **D**) and three independent experiments (**C** and **E**) are shown. Statistical significance was evaluated by *t*-test. *P* indicates the results from significance testing between the individual bile samples, fixed *E. coli*, or 5-OP-RU with the isotype control compared with the corresponding samples with the anti-MR1 antibody. All data are presented as means ± SEM (**D** and **E**). ∗*P* < 0.05, ∗∗*P* < 0.01, ∗∗∗*P* < 0.001, and ∗∗∗∗*P* < 0.0001. PBS, phosphate-buffered saline.

To investigate whether the observed MAIT cell activation was dependent on MR1 antigen–TCR interaction, TCR-mediated activation was blocked with a monoclonal anti-MR1 antibody (clone 26.5) which has previously been shown to interfere with MR1-TCR interaction.^51^ In experiments with direct incubation of bile and PBMCs, the expression of the activation markers CD69 and GrB was reduced in three of eight bile samples after blocking of the TCR with the anti-MR1 antibody (Figure 3, A and D). The MR1 restriction was not restricted to a single donor because similar blocking was seen in an experiment using one of the activating bile samples and 10 different healthy donors (Figure 3B). The MR1-independent MAIT cell activation observed for the five remaining bile samples could be explained by cytokine stimulation with multiple possible sources due to the mix of mononuclear cells in the PBMCs used in the experiments.^49^^,^^52^, ^53^, ^54^ In the cell-based assay, where APCs were preincubated with bile followed by washings before adding the PBMCs, reduction of CD69 and GrB expression was seen for all five bile samples that activated the MAIT cells (Figure 3, C and E), implying involvement of the MR1-TCR pathway for all five bile samples.

### Bile from Patients with Non-PSC Chronic Liver Diseases Activates MAIT Cells

**Figure 4:**
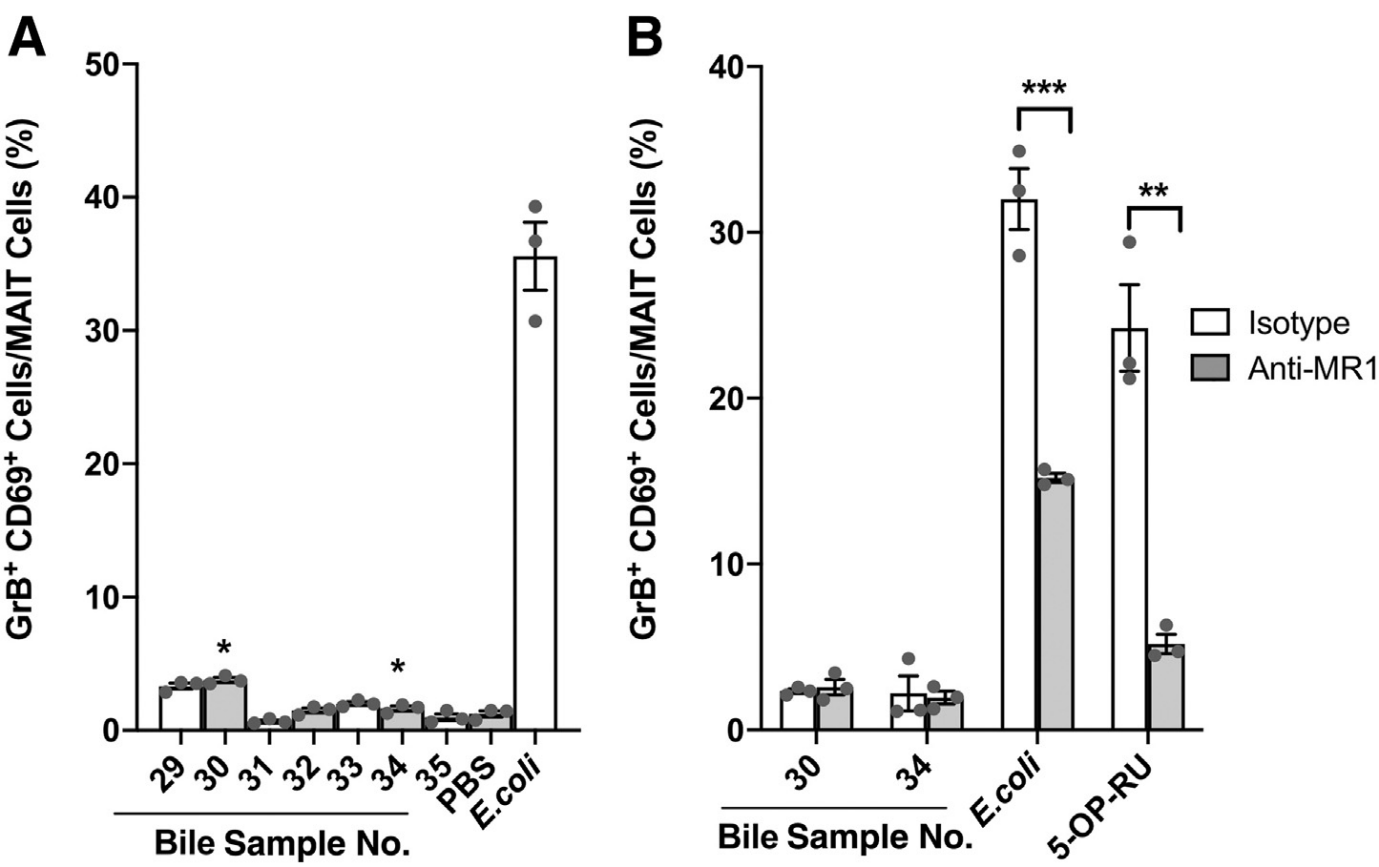
Mucosal-associated invariant T (MAIT) cells are activated by antigens in bile from patients with other chronic liver diseases than primary sclerosing cholangitis (PSC). Bile from seven patients with other chronic liver diseases than PSC was used to evaluate if activation of MAIT cells by biliary antigens was specific to bile from patients with PSC. **A:** Bar plots showing the percentage of CD69^+^ granzyme B^+^ (GrB^+^) MAIT cells after incubation with the seven bile samples (diluted 1:200) that were collected from the gallbladder of the patients, at the time of liver transplantation. Fixed *Escherichia coli* is used as positive control, and phosphate-buffered saline (PBS) is used as negative control. **B:** Bar plots showing the percentage of CD69^+^ GrB^+^ activated MAIT cells after incubation with an anti–major histocompatibility complex class I–related protein (MR1) antibody (26.5) or an isotype control within peripheral blood mononuclear cells. All experiments were performed in triplicates. **A** and **B:** Representative results from one of three independent experiments with three different healthy donors are shown. Statistical significance was evaluated by *t*-test. *P* indicates the results from significance testing between individual bile samples compared with PBS. All data are presented as means ± SEM (**A** and **B**). **A** and **B:***n* = 4 patients with alcohol-related liver disease; *n* = 1 patient with hemochromatosis; *n* = 2 patients with autoimmune hepatitis. ∗*P* < 0.05, ∗∗*P* < 0.01, and ∗∗∗*P* < 0.001. 5-OP-RU, 5-(2-oxopropylideneamino)-6-d-ribitylaminouracil.

After having demonstrated that bile from patients with PSC contained antigens capable of activating MAIT cells, the study explored whether the presence of MAIT antigens in bile was specific to patients with PSC. Bile from seven patients was included with other chronic liver diseases, alcohol-related liver disease (*n* = 4), hemochromatosis (*n* = 1), and autoimmune hepatitis (*n* = 2) (Table 2), and screened for MAIT cell activating antigens in a plate assay with PBMCs. Two of the seven bile samples activated MAIT cells, as measured by increased CD69 and GrB expression: one from a patient with alcohol-related liver disease and one from a patient with autoimmune hepatitis (Figure 4A). However, the activation was more potent in the group of bile samples from patients with PSC compared with patients not with PSC. None of the potential MAIT cell activating antigens in the two MAIT cell activating bile samples could be presented by antigen-presenting cells (THP1) (data not shown), and the activation observed in the assay with PBMCs was not MR1-dependent (Figure 4B).

**Table 2:** Clinical Characteristics of Bile Sample Donors with Other Chronic Liver Diseases than Primary Sclerosing Cholangitis

								Variables at time of transplantation			
BileSample no.	Sex	Diagnosis	MELD-Na score	Liver cirrhosis	AB	Comorbidities	ERC	Bilirubin,mg/dL	ALT,U/L	ALP,U/L	CRP,mg/L
29	M	HCM	27	Yes	No		No	20.3	40	130	17
30	F	AIH	18	Yes	No		No	3.1	56	106	13
31	F	AIH, HCC	8	Yes	Yes	Splenectomy	No	1.0	23	95	12
32	M	ALD	12	Yes	No	DM, pancreatitis, endocarditis	No	1.3	48	255	8.9
33	M	ALD	17	Yes	No	DM, MI, AF, renal insufficiency	No	0.9	25	205	37
34	M	ALD	15	Yes	No		No	1.9	13	86	35
35	M	ALD	17	Yes	Yes	MI	No	1.8	22	111	36

The presence of liver cirrhosis in the explanted liver was evaluated by a liver pathologist. Biochemistry values represent the values just before liver transplantation, and whether ERC had been performed the last 6 months before liver transplantation was registered.

F, female; M, male; AB, antibiotics; AF, atrial fibrillation; AIH, autoimmune hepatitis; ALD, alcohol-related liver disease; ALP, alkaline phosphatase; ALT, alanine aminotransferase; CRP, C-reactive protein; DM, diabetes mellitus; ERC, endoscopic retrograde cholangiography; HCC, hepatocellular carcinoma; HCM, hemochromatosis; MELD-Na, Model for End-Stage Liver Disease Sodium; MI, myocardial infarction.

### Microbiome Profiling Reveals Bacteria with the Ability to Synthesize MAIT Antigens

**Figure 5:**
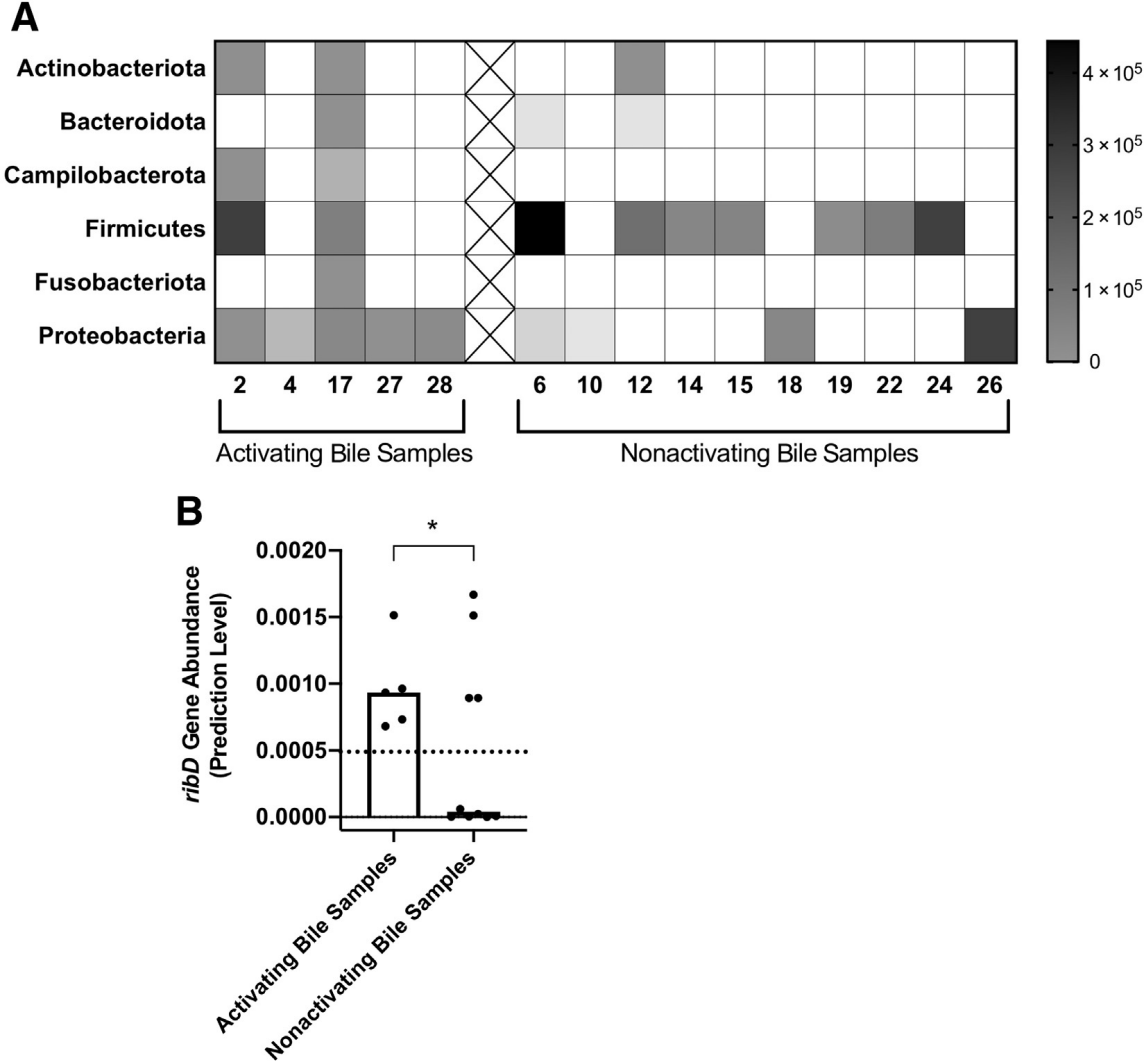
Microbiome profiling reveals bacteria with the ability to synthesize mucosal-associated invariant T (MAIT) cell antigens. **A:** The number of sequenced reads of microbial DNA at phylum level is shown for each of the 15 bile samples where microbial DNA could be detected by 16S rRNA sequencing. **B:** Prediction of the *ribD* gene abundance arranged according to MAIT cell activation in bile samples, obtained with PICRUSt2. **B:** Statistical significance was evaluated by a two-sided Fisher exact test, and the threshold for presence of *ribD* encoding bacteria was set at 0.0005 (**dashed line**). *P* indicates the results from significance testing between the presence of *ribD* encoding bacteria in the group of MAIT cell-activating and non-MAIT cell-activating bile samples. ∗*P* < 0.05.

**Supplemental Figure S5:**
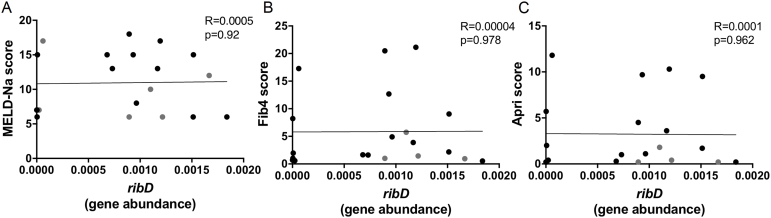
Correlation between *ribD* gene abundance and primary sclerosing cholangitis clinicopathologic staging represented by three different indexes. **A:** Model for End-Stage Liver Disease Sodium (MELD-Na) score distribution. **B:** Fibrosis-4 (Fib4) Index for liver fibrosis distribution. **C:** Aspartate Aminotransaminase to Platelet Ratio Index (APRI). Statistical significance was evaluated by Pearson *r* test. **Black lines** indicate linear regression.

To examine whether the MAIT cell-activating antigens were of microbial origin, microbial DNA was extracted and 16S rRNA sequencing of the 35 bile samples included in the study was performed. In total, microbial DNA was detected in 15 of 35 (42.9%) bile samples, as demonstrated by the 16S rRNA sequencing (Figure 5A). Of note, all of the 15 bile samples with bacterial colonization were from patients with PSC, and the frequency of colonization of bile (53.6%) was in line with the previously reported percentages among patients with PSC.^11^ Of the eight MAIT cell-activating bile samples, microbial DNA was detected in five, which represented all the bile samples that activated MAIT cells in an MR1-dependent pathway (Figure 5A). To investigate whether the sequenced bacteria in the MAIT cell-activating samples were capable of producing MAIT cell antigens (ie, vitamin B metabolites), abundance of the *ribD* gene, encoding one of the key enzymes in the vitamin B metabolism, was investigated. By comparing the sequenced bacteria against a published gene database,^48^ a higher abundance of the *ribD* gene expression in the group of MAIT cell activating bile samples compared with the group of bile samples unable to activate the MAIT cells (Figure 5B) was predicted. As expected, among the patients with PSC included in the study, 75% had concomitant IBD,^1^ but no correlation was found between IBD status and *ribD* levels or between IBD and MAIT cell-activating antigens in bile (data not shown). There were no associations between *ribD* abundance and disease severity, as measured with the clinicopathologic scores: Model for End-Stage Liver Disease Sodium score, Aspartate Aminotransaminase to Platelet Ratio Index test, and Fibrosis-4 Index (Supplemental Figure S5).

## Discussion

The combination of clinical observations and genetic studies has established PSC as a disease with features of autoimmunity, and increasing amounts of evidence point toward a role for the microbiota in the pathophysiology in PSC. However, mechanistic understanding of the interaction between the microbiota and immune system is largely lacking. This study proposes the interaction of MAIT cells with bacterial metabolites in bile as a novel pathophysiological pathway linking PSC development with the biliary microbiota.

The portal vein draining the intestine supplies the liver with antigen-rich blood and microbial products. The bile ducts represent another possible entry route for microbial products to the liver as they are in direct connection with the intestine and hence its bacterial flora. The portal tracts thus represent a seat of interaction between the microbiome and the immune system as this is where the bile ducts colocalize with the portal veins that drain into the sinusoids that are rich in different immune subsets. MAIT cells have preference to localize around the bile ducts in the portal tracts^30^^,^^32^ and are likely to interact with the microbiome at this location. In line with this, a recent publication investigating the immune cell populations in brush samples from the bile ducts detected a mucosal MAIT cell population in both PSC patients and non-PSC patients.^55^ In the present study, MAIT cells were activated by 8 of 28 bile samples from patients with PSC, suggesting a role in regulating the immune response against bile-derived pathogens. This function was not exclusive to patients with PSC as bile from patients with other end-stage liver diseases also could activate MAIT cells. The overall activation induced by non-PSC bile samples was less potent and, more importantly, the activation was not dependent on MR1-restricted antigens. These findings are in line with previous studies indicating that bile from patients with PSC is colonized with bacteria and therefore more likely contains MAIT cell antigens.^11^ IBD status was not associated with *ribD* levels or the presence of MAIT cell antigens.

The role of bile in the pathophysiology of PSC has previously been studied in the context of the toxic-bile theory, with high levels of bile salts contributing either directly or indirectly in the pathophysiology.^56^ Bile is secreted by hepatocytes with subsequent modification by cholangiocytes and consists of endogenous components such as bile salts, bilirubin, and phospholipids, as well as exogenous drugs, xenobiotics, and environmental toxins.^57^ A report indicates that bile can also contain antigens that activate natural killer T cells.^44^ The present study expands on this and shows that other major type of unconventional T cells are also activated by antigens in bile. TCR-independent modes of activation of the immune system by bile has been reported (for instance, by hepatocyte-derived IL-7 production).^25^ This mechanism could account for the partial MR1-independent activation observed for five of the eight activating bile samples. Taken together, these results establish bile as an immune active compartment of the human liver with broad ramifications.

MAIT cells possess a highly conserved semi-invariant T-cell receptor. Three different healthy donors largely showing similar results were used, suggesting that the effects observed were ubiquitous and not related to a specific donor. A MAIT cell-specific up-regulation of CD69 and GrB was observed compared with the remaining T-cell population, and blocking with an anti-MR1 antibody significantly reduced the activation. Together, these two observations strongly suggest that the effects observed are MAIT-specific and MR1-restricted. Because remaining activation was also seen after blocking MR1, it is likely that other immune-activating compounds exists in the bile, as previously reported.^44^

A healthy biliary tract has generally been considered a sterile environment, but recent evidence points toward a healthy bile microbiome.^58^ Bacteria has been detected in 40.5% to 46% of bile samples from patients with PSC, including *ribD* gene containing bacteria, such as *Klebsiella* species.^9^^,^^11^ In line with these previous findings, the study detected microbial DNA in 15 of 28 bile samples from PSC patients, including species belonging to the phyla Actinobacteriota, Bacteroidota, Firmicutes, Fusobacteriota, Campilobacterota, and Proteobacteria.^11^ Bacteria described to synthesize riboflavin-derived metabolites and potently activate MAIT cells through TCR-mediated activation^59^ overlapped with the detection of *Streptococcus*, *Klebsiella*, and *Enterobacteriaceae* in the bile samples that elicited MAIT cell activation. This was supported by a predicted higher abundance of the *ribD* gene in the group of MAIT cell activating bile samples, together providing strong evidence for the existence of microbial-derived MAIT cell activating antigens in bile. Some of these bacteria can potentially be altered with antibiotics selectively affecting some of the species known to produce MAIT antigens.^60^ These data provide a mechanistic link between the immune system and the microbiome. The MR1-dependent mechanism found in the group of PSC bile samples suggests that MAIT cells could play an important role in the pathophysiology of PSC and have implications for treatment.
